# The Application of Combined Immune Checkpoint Inhibitor Modalities in Previously Treated Non-Small Cell Lung Cancer Patients and the Associations Thereof With the Lung Immune Prognostic Index

**DOI:** 10.3389/fonc.2021.690093

**Published:** 2021-06-04

**Authors:** Ting Zhang, Xue Yang, Jing Zhao, Lixia Xia, Qiyuan Wang, Rui Jin, Lingxiao Zhou, Bin Zhang, Jun Zhao, Huijie Li, Wen Li, Yang Xia

**Affiliations:** ^1^ Department of Radiation Oncology, Second Affiliated Hospital of Zhejiang University School of Medicine, Hangzhou, China; ^2^ Key Laboratory of Carcinogenesis and Translational Research (Ministry of Education), Department of Thoracic Medical Oncology, Peking University Cancer Hospital and Institute, Beijing, China; ^3^ Department of Medical Oncology, Second Affiliated Hospital of Zhejiang University School of Medicine, Hangzhou, China; ^4^ Key Laboratory of Respiratory Disease of Zhejiang Province, Department of Respiratory and Critical Care Medicine, Second Affiliated Hospital of Zhejiang University School of Medicine, Hangzhou, China; ^5^ Department of Radiology, Second Affiliated Hospital of Zhejiang University School of Medicine, Hangzhou, China; ^6^ Department of Medical Oncology, Affiliated Hospital of Shandong University of Traditional Chinese Medicine, Jinan, China

**Keywords:** immune checkpoint inhibitor (ICI), anti-angiogenic therapy, chemotherapy, non-small cell lung cancer (NSCLC), lung immune prognostic index (LIPI)

## Abstract

**Background:**

Immune checkpoint inhibitor (ICI) monotherapy remains the standard of care for patients with previously treated non-small cell lung cancer. However, few reports have compared the clinical benefits of second-line ICIs alone with those of ICIs combined with other therapies, including anti-angiogenesis therapy or chemotherapy.

**Methods:**

Patients with previously treated advanced non-small cell lung cancer who received ICIs were retrospectively reviewed. The progression-free survival (PFS), overall survival, objective response rate, disease control rate, and safety were assessed. Complete blood cell counts and serum lactate dehydrogenase (LDH) levels were measured before and after ICI treatment.

**Results:**

Of 120 patients, 75 were treated with ICI monotherapy, 26 with ICIs plus anti-angiogenic therapy (ICI+A), and 19 with ICIs plus chemotherapy (ICI+C). The objective response rate was significantly higher in the ICI+C group (57.9%) than ICI monotherapy (26.3%) and ICI+A (31.8%) groups. The depth of response was significantly greater in the ICI+C (-35.1%) than ICI+A (−2.04%) and ICI monotherapy (3.963%) groups. ICI+C afforded a better PFS compared with the ICI monotherapy and ICI+A groups (8.5 *vs*. 4.6 and 4.1 months, respectively). Notably, the pre- and post-treatment peripheral neutrophil/lymphocyte ratios and serum LDH levels were negatively correlated with the PFS of the entire cohort. More importantly, the pretreatment lung immune prognostic index (neutrophil/lymphocyte ratio ≥ 4 and LDH level ≥ upper limit of normal) satisfactorily predicted the responses to ICI-based strategies. Adverse events (AEs) occurred in 65.3%, 92.3%, and 94.7% of patients in the ICI monotherapy, ICI+A, and ICI+C groups, respectively. Grade 3–5 AEs were more common in the combination therapy groups (ICI+A, 19.2%; ICI+C, 21%; ICI monotherapy, 4%).

**Conclusion:**

In second-line settings and beyond, ICIs combined with chemotherapy prolonged survival, with tolerable AEs. Addition of anti-angiogenic agents to ICIs did not afford any additional benefits. Further prospective studies are warranted.

## Introduction

Immunotherapy has become the new paradigm for treatment of non-small-cell lung cancer (NSCLC) (from beginning to end) and serves as an important addition to the treatment armamentarium. Monotherapy targeting the programmed death receptor 1 (PD-1) inhibitor or its ligand PD-L1 is the recommended standard of care for patients with previously treated advanced NSCLC (1, 2); such treatment significantly prolongs overall survival (OS) and exhibits a better benefit-to-risk profile compared with docetaxel chemotherapy (3). However, an initial rapid decrease in survival curves, limited objective response rates (ORRs) in entire cohorts, and the poor efficacy toward and risk of hyperprogressive disease in patients with driver gene mutations restrict the applications of immune monotherapies in clinical settings (4–8). Moreover, biomarkers of the response to second-line immunotherapy remain unclear. Currently, PD-L1 expression serves as an inclusion criterion for second-line trials. However, some of the patients who benefited lacked PD-L1 expression (9). Thus, identification of biomarkers other than PD-L1 in patients likely to respond to second-line immune checkpoint inhibitor (ICI) therapy is critical. A previous study devised a lung immunoprognostic index (LIPI), based on a neutrophil-to-lymphocyte ratio (NLR) greater than 3 and a lactate dehydrogenase level (LDH) greater than the upper limit of normal (ULN). The LIPI is an economical, rapid, and easily calculated biomarker predicting the outcomes of ICI-treated patients with advanced and emerging locally advanced NSCLC (10).

Accumulating evidence has confirmed that, in NSCLC patients, the combination of PD-1 inhibitors and chemotherapy in first-line settings improves OS and progression-free survival (PFS) more so than chemotherapy alone (11). In second-line settings, the recent PROLUNG study found that the combination of pembrolizumab and docetaxel was well-tolerated and substantially improved the outcomes of patients with advanced NSCLC, including epidermal growth factor receptor (EGFR)-mutant NSCLC (12). Preclinical and pilot clinical studies have shown that anti-angiogenic drugs, such as bevacizumab and the small molecular agents apatinib and anlotinib, potentiated the efficacy of PD-1 inhibitors by affecting the tumor environment (13).

We performed a multicenter, retrospective study to determine whether the addition of platinum-based chemotherapy or anti-angiogenic agents to PD-1 inhibitors improved the outcomes of patients with previously treated advanced NSCLC. We compared PD-1 inhibitor monotherapy with PD-1 inhibitor plus chemotherapy (ICI+C) or PD-1 inhibitor plus an anti-angiogenic agent (ICI+A) combination therapy. We also explored the predictive value of the LIPI in these contexts.

## Materials and Methods

### Patient Selection

Patients with NSCLC who received second-line immunotherapy between June 1, 2017 and March 1, 2020 at the Second Affiliated Hospital, Zhejiang University School of Medicine and Peking University Cancer Hospital were screened retrospectively. The inclusion criteria were (a) histologically confirmed unresectable stage III or IV NSCLC, (b) treatment with ICI monotherapy, ICI+C, or ICI+A as second-line or later therapy, (c) immunotherapy-naïve status, (d) a minimum of two cycles of therapy, and (e) at least one measurable lesion as defined by the Response Evaluation Criteria in Solid Tumors, ver. 1.1. The data were collected and censored to March 2021. This retrospective study was approved by the Ethics Committee of the Second Affiliated Hospital and was conducted according to the principles of the Declaration of Helsinki of 2013. The need for informed patient consent was waived by the committee given the retrospective nature of the study.

### Data Collection and Response Assessment

Complete blood cell counts and LDH levels pretreatment (i.e., within 3 days before the first treatment) and post-treatment (i.e., at 6 weeks after the first treatment) were extracted from electronic medical records. Demographic, clinical, pathological, and molecular data were also collected. The NLR was computed manually. LIPI scores were calculated based on the NLR (> 4 = 1 point) and the LDH level (> UNL = 1 point), with good, intermediate, and poor LIPI scores defined as 0, 1, and 2, respectively.

We measured the ORR, disease control rate (DCR), PFS, and OS. Patients were followed-up using computed tomography or magnetic resonance imaging until disease progression occurred. The best response was defined as a complete response or a partial response achieved at least once throughout the course of therapy, as assessed by dedicated radiologists in each center using the Response Evaluation Criteria in Solid Tumors, ver. 1.1. Toxicity data were obtained from medical records and telephone interviews during follow-up and were graded using the National Cancer Institute Common Terminology Criteria for Adverse Events, ver. 5.0.

### Statistical Analysis

Comparisons were performed using the χ2 or Fisher’s exact test for discrete variables and the unpaired t-test, Wilcoxon sign-ranked test, or analysis of variance for continuous variables. Survival curves were generated using the Kaplan–Meier method and compared using the log-rank test. Hazard ratios were calculated using Cox’s proportional hazards models. Multivariate models were used to explore the associations between biomarker levels and survival.

## Results

### Patient Demographic and Baseline Characteristics

From June 1, 2017 to March 1, 2021, 120 patients with previously treated NSCLC who received ICI monotherapy, ICI+A, or ICI+C were reviewed in terms of eligibility. Of the 120 patients, 92 (76.7%) were men, 36 (30%) were never-smokers, and 13 (10.8%) harbored EGFR or anaplastic lymphoma kinase mutations. Regarding treatment, 75 patients received ICI monotherapy, 26 ICI+A, and 19 ICI+C. All baseline characteristics including sex, age, smoking status, performance status, stage, and the treatment stage (second-line or beyond) were well-balanced among the three groups (**Table 1**). Adenocarcinomas affected 37 patients (49.3%) in the ICI monotherapy group, 11 (57.9%) in the ICI+A group, and 18 (69.2%) in the ICI+C group (**Table 1**). ICI, chemotherapy and anti-angiogenesis agents employed in the trial were listed in **Table 2**.

**Table 1:** Patient characteristics.

Characteristic	ICI monotherapy (N = 75)	ICI +Chemotherapy (N = 19)	ICI +Antiangiogenic therapy (N = 26)	P value
**Median age, years (range)**	62 (26-82)	64 (49-85)	60 (26-85)	0.271
<65 years	52 (69.3%)	12 (63.2%)	16 (61.5%)	0.672
≥65 years	23(30.7%)	7 (36.8%)	10 (38.5%)	
**Sex, n (%)**				0.320
Male	60 (80.0%)	12 (63.2%)	20 (76.9%)	
Female	15 (20.0%)	7 (36.8%)	6 (23.1%)	
**Tumor histology, n (%)**				0.086
Squamous	38 (50.7%)	7 (36.8%)	8 (30.8%)	
Adenocarcinoma	37 (49.3%)	11 (57.9%)	18 (69.2%)	
Others	0(0.0%)	1 (5.3%)	0 (0.0%)	
**Smoking history, n (%)**				0.583
Former	48 (64.0%)	10 (52.6%)	14 (53.8%)	
current	7 (9.3%)	1 (5.3%)	4 (15.4%)	
Never	20 (26.7%)	8 (42.1%)	8 (30.8%)	
**Performance status (ECOG), n (%)**				
0	1 (1.3%)	3 (15.8%)	1 (3.8%)	0.286
1	69 (92.0%)	10 (52.6%)	24 (92.3%)	
2	4 (5.3%)	6 (31.6%)	1 (3.8%)	
**Stage**				
III	15(20.0%)	4 (21.1%)	6 (23.1%)	0.946
IV	60(80.0%)	15 (78.9%)	20 (76.9%)	
**EGFR/ALK mutations**	7(9.3%)	2 (10.5%)	4 (15.4%)	0.105
**Previous systemic therapy**				
Chemotherapy	74(98.7%)	17 (89.5%)	25 (96.2%)	0.091
EGFR TKI	6(8.0%)	6 (31.6%)	4 (15.4%)	0.024
Anti-angiogenesis therapy	18(24.0%)	8 (42.1%)	8 (30.8%)	0.278
**No. of previous systemic treatments**				
1	48 12(64.0%)	12 (63.2%)	15 (57.7%)	0.829
≥2	27 (36.0%)	7 (36.8%)	11 (42.3)	
**Metastatic site**				
Brain	9(12.0%)	4 (21.1%)	1 (3.8%)	0.210
Liver	6(8.0%)	1 (5.3%)	3 (11.5.%)	0.798
Bone	19(25.3%)	3 (15.8%)	3 (11.5.%)	0.285
Lung	32(42.7%)	9 (47.4%)	13 (50%)	0.835
Pleura	18(24.0%)	3 (15.8%)	9 (34.6%)	0.335

**Table 2:** The summary of ICI, chemotherapy and anti-angiogenesis agents.

Characteristic	ICI monotherapy (N = 75)	ICI +Chemotherapy (N = 19)	ICI +Antiangiogenic therapy (N = 26)
**ICI, n (%)**			
Nivolumab	30(40%)	1(5.3%)	2 (7.6%)
Pembrolizumab	10(13.3%)	4(21.1%)	1 (3.8%)
Camrelizumab	3(4%)	9(47.3%)	17 (65.4%)
Tislelizumab	18(24%)		6(23.1%)
Sintilimab	10(13.3%)	5(26.3%)	
Atezolizumab	4(5.3%)		
**Chemotherapy drugs, n (%)**			
Paclitaxel based		13(68.4%)	
Gemcitabine		3(15.8%)	
Pemetrexed		3(15.8%)	
**Anti-angiogenesis agents, n (%)**			
Bevacizumab			3(11.5%)
Apatinib			14(53.8%)
Arotinib			3(11.5%)
Sitravatinib			6(23.1%)

### Responses to Immunotherapy

**Figure 1:**
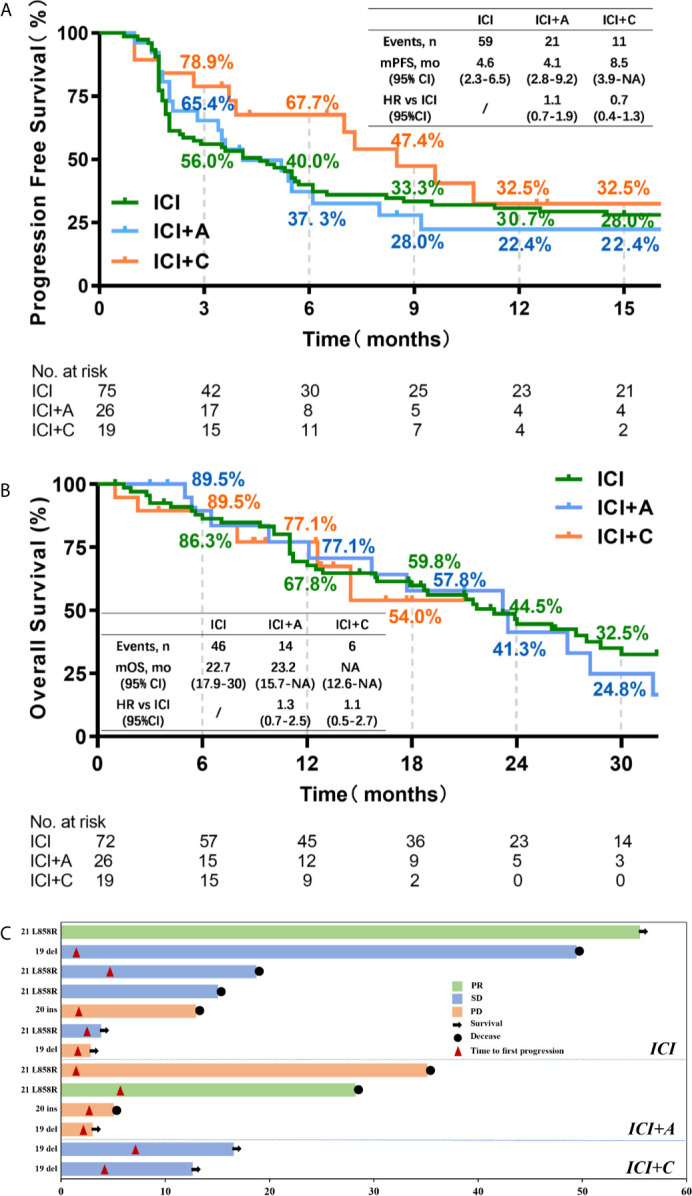
The progression-free survival and overall survival of patients treated with three immune checkpoint inhibitor-based strategies. Kaplan–Meier estimates of the progression-free survival **(A)** and overall survival **(B)** of patients treated with ICI monotherapy (green), ICI plus an anti-angiogenic agent (blue), and ICI plus chemotherapy (orange). Censored data are indicated by ticks. In the analysis of progression-free survival, data from patients who had not progressed and were still alive at the time of analysis were censored at their last assessment. In the analysis of overall survival, data from patients who were considered to be alive at the time of analysis were censored at the last recorded date on which the patients were known to be alive. **(C)** A swimmer plot summarizing the responsiveness of thirteen patients with EGFR mutations. CI, confidence interval; HR, hazard ratio; ICI, immune checkpoint inhibitor; A, anti-angiogenic therapy; C, chemotherapy; PR, partial response; SD, stable disease; PD, progressive disease.

Dedicated radiologists and physicians independently reviewed all clinical information. The median PFS times were 4.6, 4.1, and 8.5 months in the ICI, ICI+A, and ICI+C groups, respectively (**Figure 1A**). PFS tended to be longer in the ICI+C group, but the OS did not differ among the ICI monotherapy, ICI+A, and ICI+C groups (22.7, 23.2, and not attained, respectively; **Figure 1B**). A swimmer plot summarizing the responsiveness of EGFR mutant patients was shown in **Figure 1C**.

**Figure 2:**
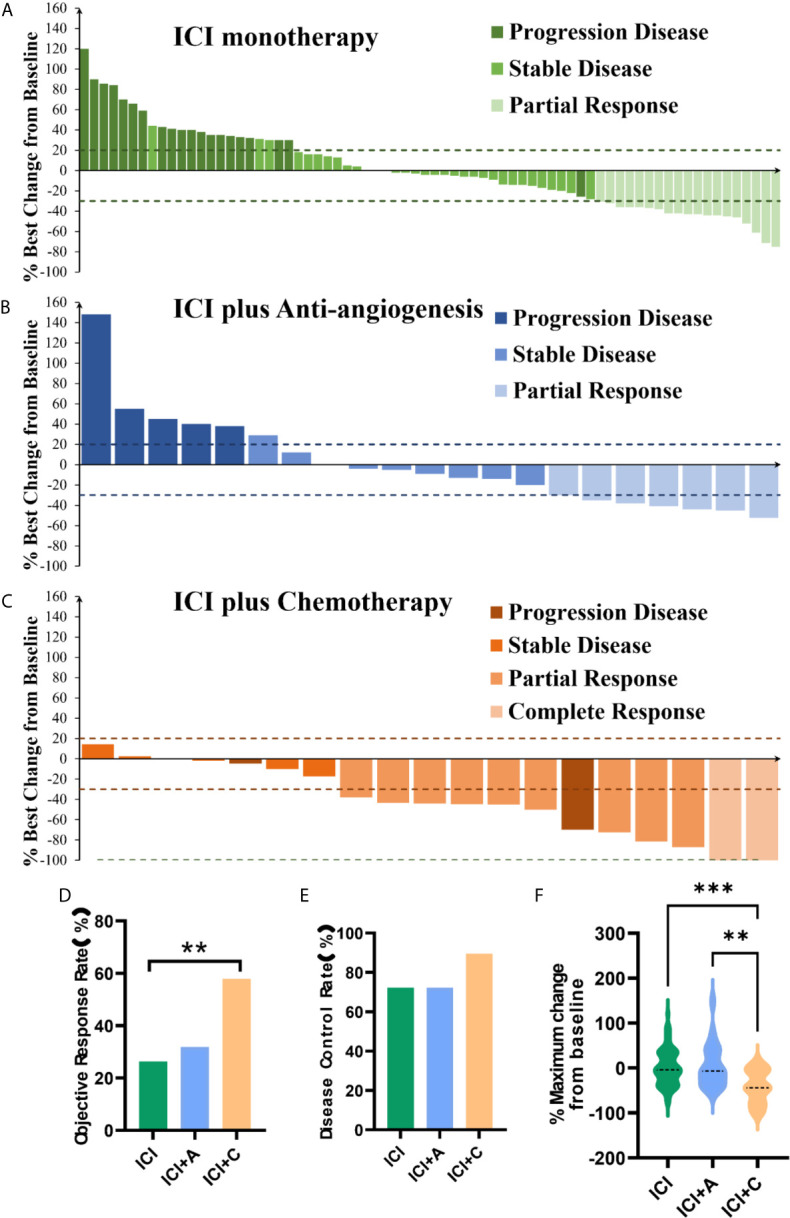
Responses to treatment. Waterfall plots of the treatment response in terms of the greatest change in tumor size compared with the pretreatment size **(A–C)** in patients treated with ICI monotherapy (green panel), ICI plus an anti-angiogenic agent (ICI+A, blue panel), and ICI plus chemotherapy (ICI+C, orange panel). Each bar represents the greatest reduction in the target lesion size in an individual patient. The dashed lines show the cutoffs used to define progressive disease (≥ 20% increase) and a partial response (≥ 30% reduction). The objective response rate **(D)**, disease control rate **(E)**, and maximum change compared with pretreatment values **(F)** in the ICI monotherapy (green), ICI+A (blue), and ICI+C (orange) groups. A complete response, a partial response, stable disease, and progressive disease were estimated using the Response Evaluation Criteria in Solid Tumors criteria, ver. 1.1. ICI, immune checkpoint inhibitor; A, anti-angiogenic agent; C, chemotherapy. **P < 0.01, ***P < 0.001.

We evaluated the treatment responses. The ORR was significantly higher in the ICI+C group (57.9%) than ICI monotherapy group (26.3%) and ICI+A group (31.8%, P = 0.036, **Figure 2D**). In contrast, the DCR was similar among the three arms (ICI monotherapy *vs*. ICI+A *vs*. ICI+C: 72.2% *vs*. 72.2% *vs*. 89.5%, P = 0.275, **Figure 2E**). The depths of the treatment responses are summarized in **Figure 2**. Of patients in the ICI+C group, two with a complete response had an average depth of response of -35.1%, which was significantly greater than those in the ICI+A group (−2.04%, P = 0.0161) and ICI monotherapy group (3.963%, P = 0.0105). The percentages of patients exhibiting no reduction in tumor size in the ICI monotherapy, ICI+A, and ICI+C groups were 45.1%, 38.1%, and 11.1%, respectively.

### Associations of the NLR and LDH Level With Clinical Efficacy

**Figure 3:**
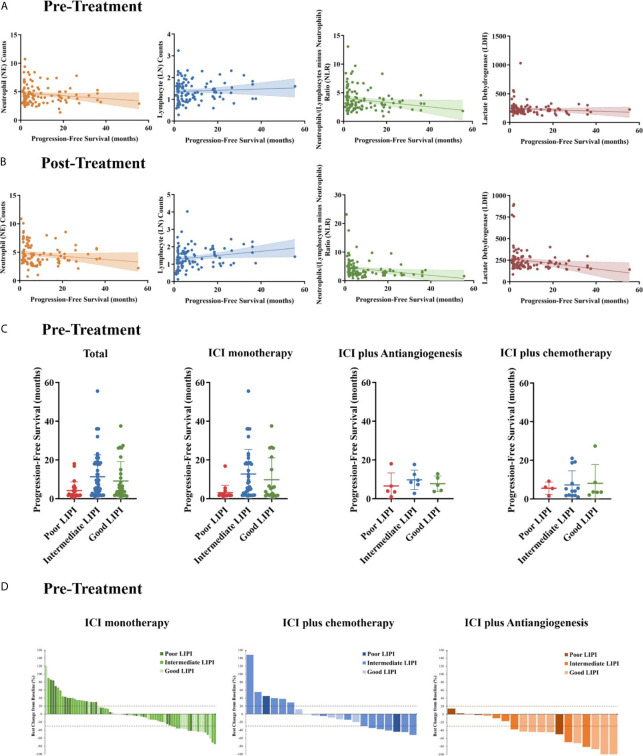
Pre- and post-treatment biomarker measurements. The associations between pre- **(A)** and post-treatment **(B)** neutrophil (NE) counts, Lymphocyte (LN) counts, neutrophil/lymphocyte ratios (NLRs), and the serum lactate dehydrogenase (LDH) level with progression-free survival (PFS). **(C)** PFS stratified by the lung immune prognostic index (LIPI) score in all patients, the ICI monotherapy group, the ICI plus anti-angiogenic agent (ICI+A) group, and the ICI plus chemotherapy (ICI+C) group. **(D)** Waterfall plots showing the best responses and the LIPI scores in the ICI monotherapy (green panel), ICI+A (blue panel), and ICI+C (orange panel) groups. The LIPI is based on an NLR greater than 3 and an LDH level greater than the upper limit of normal. ICI, immune checkpoint inhibitor.

We analyzed the associations of the peripheral absolute neutrophil count, absolute lymphocyte count (LNC), NLR, and LDH level between pretreatment and post-treatment. Therapeutic efficacy was evident in the entire cohort. Pretreatment, the LNC was positively, but the absolute neutrophil count negatively, associated with PFS, indicating a significant negative association between the pretreatment NLR and PFS (r = −0.1962, P = 0.0365, **Figure 3A**). Notably, the correlations of PFS with the LNC and NLR were more pronounced after two cycles of treatment (LNC: r = 0.2106, P = 0.0287; NLR: r = −0.2273, P = 0.0186, **Figure 3B**). Similarly, PFS was negatively associated with the pretreatment LDH level and even more so with the post-treatment level (r = −0.2312,P = 0.0182).

A pretreatment NLR greater than 4 was independently associated with PFS, and a pretreatment LDH level greater than the ULN was marginally associated with PFS in a Cox’s proportional hazard model. These two biomarkers were combined to create the LIPI, as reported previously (14). Of 113 evaluable patients, 31 (27.4%) had good LIPI scores (NLR < 4 and LDH level < ULN), 57 (50.4%) intermediate scores (NLR ≥ 4 or LDH level ≥ ULN), and 25 (22.1%) poor scores (NLR ≥ 4 and LDH ≥ ULN). The median PFSs of the patients with poor, intermediate, and good LIPI scores were 4.2, 11.3, and 9.1 months, respectively (P = 0.0119, **Figure 3C**). We generated waterfall plots of the best responses and LIPI scores (**Figure 3D**).

### Safety

**Figure 4:**
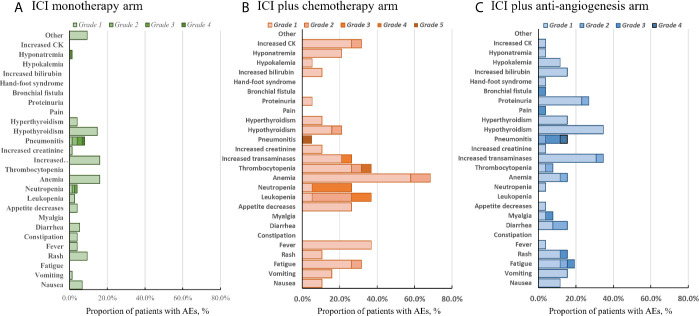
Adverse events. All-cause adverse events of grades 1–5 in the ICI monotherapy **(A)**, ICI plus chemotherapy **(B)**, and ICI plus anti-angiogenic agent **(C)** groups. The color intensity reflects severity.

The different treatment strategies were associated with unique adverse events (AEs) (**Figure 4**). During initial therapy, treatment-related AEs occurred in 65.3% of patients in the ICI monotherapy group, 92.3% in the ICI+A group, and 94.7% in the ICI+C group. Serious (grade 3–5) treatment-related AEs occurred in five-fold more patients in the combination treatment groups (ICI+A, 19.2%; ICI+C arm, 21%) than in the ICI monotherapy group (4%). The most common AEs included fever, fatigue, loss of appetite, nausea, vomiting, and diarrhea. More hematological toxicities were observed in the ICI+C group, whereas hypertension and proteinuria were more common (but not severe) in the ICI+A group. The rates of immune-related AEs (irAEs), such as thyroid dysfunction and pneumonitis, were comparable among the three groups. Notably, one patient in the ICI+C group developed grade 5 pneumonitis.

## Discussion

To the best of our knowledge, this is the first study to compare the efficacies of ICI+C, ICI+A, and ICI monotherapy in patients with previously treated NSCLC. We also evaluated the value of the LIPI score as a biomarker. We found that ICI+C treatment significantly improved the ORR and depth of response and tended to improve the PFS of previously treated patients, more so than did ICI monotherapy. Compared with ICI monotherapy, ICI+A as second-line or later therapy did not afford any additional clinical benefits in terms of the ORR, DCR, depth of response, PFS, or OS. Of note, the pre- and post-treatment peripheral NLRs and LDH levels were correlated with the PFS of the whole cohort, and more importantly, the pretreatment LIPI score well-predicted the responsiveness to ICI-based strategies in NSCLC patients undergoing second-line or later therapy.

In the first-line setting, anti-PD-1/PD-L1 monotherapy (2, 15) combined with chemotherapy (16, 17), or chemotherapy combined with bevacizumab (18), significantly improved survival, with favorable safety profiles. However, ICI monotherapy is usually recommended for the second-line setting, in which the ORR is almost 20%, PFS 3.5–4.2 months, and OS 9.2–12.2 months (1, 19, 20). In the phase 2 PROLUNG trail, compared with docetaxel monotherapy, pembrolizumab plus docetaxel improved the ORR from 15.8% to 42.5% and the modified PFS from 3.9 to 9.5 months (12). One retrospective study reported a trend of longer PFS (7.5 *vs*. 3.7 months) and a significant improvement in OS (28.6 *vs*. 15.9 months) in the ICI plus nab-paclitaxel group compared with the ICI monotherapy group. We found that the ICI monotherapy group exhibited an ORR of 26.3% and a PFS of 4.6 months. The median PFS was 9.1 months in the ICI+C group, comparable with that in the PROLUNG trial. In line with previous findings, although statistical significance was not attained, the PFS also tended to be better with combination therapy. However, the OS curves of the three groups overlapped extensively. Several studies have shown that anti-angiogenic agents act synergistically with PD-1/PD-L1 inhibitors to improve the low efficacy of ICI monotherapy, with an ORR of ~30% (21, 22). The combination treatments increased infiltration of CD8+ T cells, reduced recruitment of tumor-associated macrophages, reversed inhibition of DC maturation, and promoted the development of an angiostatic and immune system-activating tumor microenvironment (23, 24). In second-line or higher settings, a real−world retrospective study found that a PD-1 inhibitor plus anlotinib was associated with an ORR of 19.3%, DCR of 85.5%, and PFS of 5 months (25). In our present study, the ORR, DCR, and PFS of the ICI+A group were 31.8%, 72.7%, and 4.1 months, respectively. However, our data suggest that the addition of anti-angiogenic agents to ICIs does not translate into improved outcomes. To the best of our knowledge, this is the largest cohort study to compare ICI monotherapy, ICI+C, and ICI+A simultaneously. ICI+C should be considered in second-line and higher settings, but evidence supporting the combination of an anti-angiogenic agent with an ICI in patients with previously treated NSCLC is lacking.

The lack of significant differences in PFS and OS has several possible explanations. First, the proportions of patients who did not attain the PFS (47.3%) and OS (68.9%) endpoints were higher in the ICI+C group than in the other two groups. We suspect that the significant survival benefit of the ICI+C group reflects the longer follow-up period in this group. Second, different PD-1 inhibitors were used. The selection of PD-1/PD-L1 inhibitors in the real world depends on the clinical evidence, patient’s choice, and physician’s experience, all of which cause bias.

Inflammation, particularly chronic inflammation, is tightly linked to cancer progression (26). Inflammatory cytokines influence lymphocytes and neutrophils. Many routine blood parameters have been investigated as potential inflammatory biomarkers, including elevated neutrophil and LDH levels and hypoalbuminemia, all of which are associated with poor cancer outcomes (27). The pretreatment NLR is a well-known prognostic factor in patients with NSCLC (28); however, the value of the post-treatment NLR has not been fully explored. This is the first study to evaluate the effects of both pretreatment and post-treatment parameters on the outcomes of three different ICI-based treatments. Interestingly, we found that the NLR, especially the post-treatment NLR, strongly predicted the outcomes of later-line ICI-based strategies. The LDH level is a classic inflammatory marker in patients with cancer. When the tumor burden is high, an elevated LDH level reflects increased tumor glycolytic activity and tumor necrosis caused by hypoxia (29). The LDH level was inversely related to the response to ICIs and may even trigger hyperprogressive disease. We found that the pretreatment LDH level tended to have a negative association with PFS, and that the post-treatment LDH level was significantly associated with poor PFS, reflecting the potential utility of the LDH level as a biomarker.

There are some limitations in our study. First, this was a retrospective study with a relatively small sample size, and the three groups were not completely balanced. The ICI+C group comprised more patients with EGFR mutations compared with the other groups. Second, due to the retrospective nature of our study, the platforms and calculated logics of TMB were varied, also the antibody used for PD-L1 testing were different. Thus, to avoid any artificial effect, we did not analyze these validated biomarkers. Third, several ICIs were used, including nivolumab, pembrolizumab, camrelizumab, tislelizumab, and sintilimab. The effects of each drug may differ. Finally, a longer follow-up time is needed to estimate OS more objectively, especially in the ICI+C group.

In conclusion, ICI monotherapy remains the standard of care in second-line settings. ICI+C combination therapy afforded certain advantages and tolerable AEs. Although addition of an anti-angiogenic agent to an ICI should theoretically afford a synergistic effect, we failed to detect any such effect. Further prospective studies are warranted.

## Data Availability Statement

The original contributions presented in the study are included in the article/supplementary material. Further inquiries can be directed to the corresponding authors.

## Ethics Statement

The studies involving human participants were reviewed and approved by Ethics Committee of the Second Affiliated Hospital. Written informed consent for participation was not required for this study in accordance with the national legislation and the institutional requirements.

## Author Contributions

TZ, XY, JiZ, LX, HL, WL and YX contributed to study conception and design. JiZ, LX, QW, LZ, BZ and YX conducted data collection. TZ, XY, JiZ, LX, QW, RJ, LZ, BZ, JuZ and YX conducted data analysis. TZ, XY, JiZ, LX, QW and YX drafted the manuscript. All authors contributed to the article and approved the submitted version.

## Funding

This work was supported by the National Natural Science Foundation of China [81870022] and the Zhejiang Provincial Natural Science Foundation [LY20H010004, LY21H100004].

## Conflict of Interest

The authors declare that the research was conducted in the absence of any commercial or financial relationships that could be construed as a potential conflict of interest.
